# Clinical outcomes of microdissection testicular sperm extraction and intracytoplasmic sperm injection in Japanese men with Y chromosome microdeletions

**DOI:** 10.1002/rmb2.12317

**Published:** 2020-01-13

**Authors:** Kohei Yamaguchi, Tomomoto Ishikawa, Shimpei Mizuta, Takumi Takeuchi, Hidehiko Matsubayashi, Shoji Kokeguchi, Toshihiro Habara, Kentaro Ichioka, Masakazu Ohashi, Sumihide Okamoto, Toshihiro Kawamura, Satoru Kanto, Hisanori Taniguchi, Fumiko Tawara, Tetsuaki Hara, Hatsuki Hibi, Hiroshi Masuda, Takehiko Matsuyama, Hiroaki Yoshida

**Affiliations:** ^1^ Division of Infertility Reproduction Clinic Osaka Osaka Japan; ^2^ Kyono ART Clinic Takanawa Tokyo Japan; ^3^ Hanabusa Womens Central Fertility Clinic Hyogo Japan; ^4^ Okayama Couple's Clinic Okayama Japan; ^5^ Ichioka Urological Clinic Kyoto Japan; ^6^ Department of Urology Ogikubo Hospital Tokyo Japan; ^7^ Obstetrics and Gynecology ART Okamoto Women's Clinic Nagasaki Japan; ^8^ Denentoshi Ladies Clinic Kanagawa Japan; ^9^ Kantou Clinic Miyagi Japan; ^10^ IVF Namba Clinic Osaka Japan; ^11^ Tawara IVF Clinic Shizuoka Japan; ^12^ Division of Reproductive Medicine Hiroshima Prefectural Hospital Hiroshima Japan; ^13^ Division of Urology Kyoritsu General Hospital Aichi Japan; ^14^ Tesseikai Cranial Nerve Surgery Hospital Osaka Japan; ^15^ Koujin Hospital Kagawa Japan; ^16^ Sendai ART Clinic Miyagi Japan

**Keywords:** azoospermia factor, embryo transfer, intracytoplasmic sperm injection, testicular sperm extraction, Y chromosome microdeletions

## Abstract

**Purpose:**

We investigated the clinical results of Japanese men with Y chromosome microdeletions.

**Methods:**

This study retrospectively examined 2163 azoospermic or severe oligozoospermic patients. We investigated the frequency of azoospermia factor (AZF) deletions and sperm retrieval rate (SRR) by microTESE in patients with these deletions, then analyzed the ICSI outcomes.

**Results:**

Azoospermia factor deletions were found in 201 patients. SRR was significantly higher than that of the control group (74.0% vs 20.4%, *P* < .001). Thirty‐three couples underwent ICSI using testicular spermatozoa retrieved by microTESE, and eight couples underwent ICSI using ejaculatory spermatozoa. The fertilization rate and clinical pregnancy rate per embryo transfer cycle were significantly higher in the ejaculatory group than that of the testicular group (66.4% vs 43.7%, *P* < .001, 53.3% vs 24.7%, *P* = .03, respectively). When compared with the control group, the fertilization rate was significantly lower in the testicular group with AZFc microdeletions (43.7% vs 53.6%, *P* < .001).

**Conclusions:**

Our study highlights that although microTESE in azoospermic men with AZFc microdeletions led to a higher SRR, ICSI outcomes of these men were worse than that of men without AZF deletions, even if testicular spermatozoa were retrieved.

## 1 INTRODUCTION

After Klinefelter syndrome, Y chromosome microdeletions are the second most frequent genetic cause of male infertility, with a prevalence of 2%‐10% in non‐obstructive azoospermia (NOA).^1^, ^2^, ^3^ Three spermatogenesis loci in the Y chromosome long arm (Yq11) have been classified as AZFa, AZFb, and AZFc.^4^ The classical correlation of histopathology phenotypes with these three microdeletions comprises of complete absence of germ cells (Sertoli cell‐only syndrome) in patients with AZFa microdeletions, maturation arrest of meiosis in patients with AZFb microdeletions, and hypospermatogenesis in patients with AZFc microdeletions.^4^, ^5^, ^6^, ^7^, ^8^, ^9^, ^10^, ^11^ However, individual variation in the extent of deletions has led to various spermatogenic phenotypes.

Patients with azoospermia factor (AZF) deletions, especially AZFc microdeletions, have a chance to father offspring through sperm extraction techniques such as microTESE and in vitro fertilization with ICSI.^12^, ^13^, ^14^, ^15^ Stahl et al^16^ reported that, using microTESE, sperm retrieval is higher from NOA patients with AZFc microdeletions than from idiopathic azoospermic men.

Azoospermia factor deletions are associated not only with disruption of spermatogenesis, but also with ICSI outcomes. Mateu et al^17^ reported a high percentage of aneuploidies in spermatozoa and embryos from patients with Y chromosome microdeletions. In addition, both fertilization rate and embryo quality were significantly lower in patients with AZFc microdeletions, but pregnancy, implantation, and take‐home baby rates were not significantly affected.^18^ Although various findings have been reported, the phenotypic influence of AZF deletions clearly depends on the ethnic and geographic origin of the study population.^19^ In the present study, we sought to clarify the frequency of each type of AZF deletion in Japanese azoospermic or very severe oligozoospermic patients, the SRR following microTESE in these patients with AZF deletions, and the ICSI outcomes from testicular or ejaculatory spermatozoa of these patients. Furthermore, we compared these results with their respective control groups.

## 2 MATERIALS AND METHODS

A total of 2065 azoospermic and 98 very severe oligozoospermic patients were enrolled. All men suffering from infertility presented themselves to one of 16 reproductive centers in Japan from 2007 to 2017. They underwent semen analysis at least two times. After the diagnosis of azoospermia or very severe oligozoospermia (a sperm count of <1 million per mL), karyotype analysis and detection of Y chromosome microdeletions were performed on peripheral blood lymphocytes of these patients.

### 2.1 Y chromosome microdeletions

Y chromosome microdeletions in AZFa, AZFb, and AZFc regions were detected using Promega Y Chromosome AZF Analysis System version 2.0 following the manufacturer's instructions (Promega Co.). Twenty sequence‐tagged sites within the AZF region of Yq11 and the sex‐determining region Y gene were targeted for polymerase chain reaction (PCR) amplification. This system covers all of the loci recommended by the European Academy of Andrology and European Quality Monitoring Network Group. Multiplex PCR was performed for analysis of microdeletions.

### 2.2 MicroTESE procedure

If no spermatozoa were identified within the semen pellet, microTESE was performed under local anesthesia by experienced surgeons as previously described.^20^ We utilized the operating microscope and one transverse incision in the tunica albuginea through which spermatogenic tubules were selected for extraction. Almost all testicular spermatozoa retrieved by microTESE were cryopreserved for later ICSI use.

### 2.3 Controlled ovarian stimulation and allocation

Most women underwent controlled ovarian stimulation with a gonadotropin‐releasing hormone (GnRH) short antagonist or a GnRH short/long agonist protocol.^21^, ^22^ For ovarian stimulation, human menopausal gonadotropin was mainly used. Transvaginal ultrasound‐guided oocyte retrieval was conducted 34‐36 hours after the stimulation. One or two embryos were frozen by vitrification on the third day following oocyte retrieval, and the other embryos were placed in extended culture, from which blastocysts were frozen on day 5 or day 6.

### 2.4 Endometrium preparation and embryo transfer

Hormone replacement therapy was employed for endometrium preparation in most cases. Briefly, oral ethinyl estradiol was administered from day 3 to attain an endometrial thickness ≥7‐8 mm. At that time, patients were given luteal supplementation via intravaginal and/or oral administration of progesterone. Freeze‐thawed ET (FET) of day 3 embryos or day 5 or day 6 blastocysts were scheduled based on the embryo and endometrium synchronization. Clinical pregnancy was established by ultrasonography at 5 or 6 weeks of gestation. Oral estradiol and progestin were continued until 10 weeks of gestation when pregnant.

### 2.5 Statistical analysis

The data are presented as the mean ± standard deviation. Mann‐Whitney *U*‐test was used for continuous data and Fisher's exact test was used for categorical data to evaluate comparisons between the groups. A *P* value <.05 was regarded as significant.

## 3 RESULTS

### 3.1 Incidence of AZF microdeletions in the Japanese population

One hundred and eighty‐four AZF microdeletions were detected in the azoospermic patients, and 17 microdeletions in patients with very severe oligozoospermia. The number of patients found to have each AZF deletion is shown in Table 1. All men with AZFa, AZFb, AZFa + b, and AZFb + c microdeletions in Yq11 had azoospermia. The 17 men with AZFc microdeletions had very severe oligozoospermia. The most common deleted region was AZFc (3.7%). Among the entire cohort, we performed subsequent examinations on cases in which the details of treatment were confirmed.

**Table 1:** Prevalence of Y chromosome microdeletions

Diagnosis from semen analysis	No. of screened patients	No. of patients with deletions	No. of patients in each deletion group					
			AZFa	AZFb	AZFc	AZFa + b	AZFb + c	Yq (AZFa + b + c)
Azoospermia	2065	184	20	15	64	1	61	23
Severe oligozoospermia	98	17	0	0	17	0	0	0
Total no. of patients (%)	2163	201 (9.3)	20 (0.9)	15 (0.7)	81 (3.7)	1 (0.05)	61 (2.8)	23 (1.1)

AZF, azoospermia factor.

### 3.2 SRR in AZF deletions

Fifty azoospermic men with AZFc microdeletions underwent microTESE, and spermatozoa were detected in 74% (37/50) of these men. In contrast, we detected spermatozoa in only 20.4% (109/534) of the azoospermic men without AZF deletions. The characteristics and SRR of each group are shown in Table 2. The SRR was much higher in patients with AZFc microdeletions than that of patients without AZF deletions. Although three azoospermic men with AZFb + c microdeletions had also undergone microTESE following patient request, we did not detect spermatozoa.

**Table 2:** Clinical characteristics of azoospermic patients and outcomes of microTESE

	AZFc microdeletions (n = 50)	No AZF deletions (n = 534)	*P*‐value
Age (y.o.)	35.1 ± 5.0	34.0 ± 5.5	.08*
Testicular volume (mL)	12.1 ± 4.5	12.0 ± 4.1	.63*
FSH (mIU/mL)	20.4 ± 10.4	19.9 ± 9.8	.72*
LH (mIU/mL)	7.1 ± 4.2	6.8 ± 3.7	.91*
Testosterone (ng/mL)	4.2 ± 1.6	4.6 ± 1.8	.10*
Sperm retrieved rate (%)	74.0 (37/50)	20.4 (109/534)	<.001**

AZF, azoospermia factor.

* Mann‐Whitney *U*‐test.

** Fisher's exact test.

### 3.3 ICSI outcomes

Thirty‐three couples underwent ICSI using testicular spermatozoa retrieved by microTESE, and eight couples underwent ICSI using ejaculatory spermatozoa. In this study, no couple underwent ICSI using both testicular and ejaculatory spermatozoa. The outcomes of ICSI are shown in Table 3. The fertilization rate was 47.6% (291/611). We performed 92 embryo transfer (ET) cycles, and the mean number of replaced embryos was 1.42 per ET. The clinical pregnancy rate per ET cycle was 29.3% (27/92). The abortion rate was 22.2% (6/27). The fertilization rate was significantly higher in the ejaculatory group than in the testicular group (66.4% vs 43.7%, *P* < .001). The clinical pregnancy rate per ET cycle was also significantly higher in the ejaculatory group (53.3% vs 24.7%, *P* < .03).

**Table 3:** ICSI outcomes of couples with AZFc microdeletions

	Total (n = 41)	Testicular sperm (n = 33)	Ejaculated sperm (n = 8)	*P*‐value
Maternal age (y.o.)	33.9 ± 4.2	34.0 ± 4.2	33.9 ± 4.5	.61*
Oocyte retrieval cycles	86	67	19	
Two PN (%)	47.6 (291/611)	43.7 (220/504)	66.4 (71/107)	<.001**
ET cycles	92	77	15	
Embryos replaced (n)	1.42 ± 0.56	1.40 ± 0.57	1.53 ± 0.50	.38*
Clinical pregnancies/cycle (%)	29.3 (27/92)	24.7 (19/77)	53.3 (8/15)	.03**
Miscarriage (%)	22.2 (6/27)	26.3 (5/19)	12.5 (1/8)	.63**

Abbreviations: AZF, azoospermia factor; ET, embryo transfer; PN, pronucleaous.

* Mann‐Whitney *U*‐test.

** Fisher's exact test.

The two groups were also compared with their respective control groups. The fertilization rate was significantly lower in the testicular group than in the control group (218 ICSI treatments in 105 couples comprised of men without AZF deletions; 43.7% vs 53.6%, *P* < .001; Table 4). In contrast, there were no significant differences in any parameters of the ejaculatory group compared with the control group of 109 ICSI treatments in 46 couples consisting of men without AZF deletions (Table 5).

**Table 4:** ICSI outcomes using testicular sperm

	AZFc microdeletions (n = 33)	No AZF deletions (n = 105)	*P*‐value
Maternal age (y.o.)	34.0 ± 4.2	34.9 ± 3.9	.09*
Oocyte retrieval cycles	67	218	
Two PN (%)	43.7 (220/504)	53.6 (958/1787)	<.001**
ET cycles	77	291	
Embryos replaced (n)	1.40 ± 0.57	1.34 ± 0.57	.40*
Clinical pregnancies/cycle (%)	24.7 (19/77)	28.9 (84/291)	.57**
Miscarriage (%)	26.3 (5/19)	20.2 (17/84)	.55**

Abbreviations: AZF, azoospermia factor; ET, embryo transfer; PN, pronucleaous.

* Mann‐Whitney *U*‐test.

** Fisher's exact test.

**Table 5:** ICSI outcomes using ejaculated sperm

	AZFc microdeletions (n = 8)	No AZF deletions (n = 46)	*P*‐value
Maternal age (y.o.)	33.9 ± 4.5	31.9 ± 6.0	.10*
Oocyte retrieval cycles	19	109	
Two PN (%)	66.4 (71/107)	69.9 (334/478)	.49**
ET cycles	15	87	
Embryos replaced (n)	1.53 ± 0.50	1.39 ± 0.62	.35*
Clinical pregnancies/cycle (%)	53.3 (8/15)	34.5 (30/87)	.25**
Miscarriage (%)	12.5 (1/8)	20.0 (6/30)	1.00**

Abbreviations: AZF, azoospermia factor; ET, embryo transfer; PN, pronucleaous.

* Mann‐Whitney *U*‐test.

** Fisher's exact test.

## 4 DISCUSSION

In this study, the incidence of Y chromosome microdeletions in patients with NOA was 8.9% (184/2065). Other studies from various populations have reported Y chromosome microdeletions in 2%‐10% of azoospermic patients.^1^, ^2^, ^3^ In contrast, we diagnosed Y chromosome microdeletions in 17.3% (17/98) of very severe oligozoospermic men. This is high compared with the 6%‐10% reported incidence of Y chromosome microdeletions in severe oligozoospermic men that were tested and treated at other centers throughout the world.^2^, ^17^, ^23^, ^24^, ^25^ This result may reflect selection bias of our limited screened population. However, the substantial rate of Y chromosome microdeletions in very severe oligozoospermic men supports the necessity to perform screening of Y chromosome microdeletions in these men, even in cases where enough spermatozoa for assisted reproductive technology (ART) has already been collected. Failure to do so may compromise the treating physician's ability to adequately counsel these patients before ART about the risks of subfertility in their male offspring.

Spermatid arrest and even crypto/oligozoospermia have been reported in association with complete AZFb or AZFb + c microdeletions.^11^, ^26^ With very few exceptions reported in the literature, complete deletion of AZFa or AZFb clinically implies that the chance for testicular spermatozoa retrieval is virtually zero, even with microTESE.^17^, ^27^ Therefore, these patients are not recommended to undergo this procedure. In contrast, we identified testicular spermatozoa in over 70% of azoospermic patients with AZFc microdeletions. This SRR is much higher than that of non‐AZF deleted, idiopathic NOA men. The biological explanation of this result remains unclear, but a previous study of the US population reported similar results.^16^ In the present study, we compared the ICSI outcomes between spermatozoa of different origins. Although the number of cases was limited, fertilization rate and clinical pregnancy rate were significantly higher in the ejaculatory group than in the testicular group. Furthermore, when compared with their respective control group, the fertilization rate was significantly lower in the testicular group, but there were no significant differences in any parameters in the ejaculatory group. As described by Van et al,^18^ the results of our study indicate that the main function of the AZFc region in the Y chromosome is involvement in spermatozoa quality or function rather than in spermatogenesis. However, our results also indicate that the loss of spermatozoa quality associated with AZFc microdeletions may be restored by modification when passing through the epididymis.

Regardless of this hypothesis, our results revealed a number of important factors to be considered during pre‐treatment counseling of couples comprised of men with AZFc microdeletions. At first, when ejaculate spermatozoa are detected in pre‐treatment semen analyses, even if there are only small amounts of sperm, we should clinically recommend primary use of ejaculatory, rather than testicular, spermatozoa. Secondly, if the patient must undergo microTESE, we have to inform them that, although microTESE leads to a higher SRR, ICSI may lead to a lower fertilization rate relative to the control group.

To our knowledge, the present study has investigated the clinical outcomes, including microTESE and ICSI, of the largest cohort of Japanese subfertile men with Y chromosome microdeletions to date. Nevertheless, generalizing our findings may be limited. The cohort size of this study is relatively small, and, therefore, our screened population of infertile men may be biased. The high incidence of Y chromosome microdeletions, especially in patients with very severe oligozoospermia, may reflect selection bias due to our referral pattern. The ICSI or ET protocol was also not consistent among all centers. Moreover, genetic testing data from male offspring were not available, so we cannot examine the inheritance of Y chromosome microdeletions. Further investigations using a larger number of couples with AZFc microdeletions, including their male offspring, are required to study the prognosis of these deletions after ICSI treatment.

In conclusion, NOA patients with AZFc microdeletions had a high percentage of successful sperm retrieval by microTESE, but the ICSI outcomes, especially fertilization rate, were not good. Although analysis of Y chromosome microdeletions is still not offered in some centers, our study emphasizes that diagnosis of Y chromosome microdeletions is critical for preconception genetic counseling and provides clinically valuable prognostic information to couples considering surgical sperm retrieval and ICSI.

## DISCLOSURES

*Conflict of interest*: All of the authors declare that they have no conflict of interest. *Human rights statements and informed consent*: All procedures followed were in accordance with the ethical standards of the responsible committee on human experimentation (institutional and national) and with the Helsinki Declaration of 1964 and its later amendments. In all participating facilities, strict common informed consent was obtained from all patients for inclusion in the study. All patient data were collected and used following personal information protection such as anonymization. *Animal studies*: This article does not contain any studies with animal subjects performed by any of the authors. *Approval by ethics committee*: The protocol for this research project including human patients has been approved by a suitably constituted Ethics Committee.

## References

[1] Krausz C , Rajpert‐De Meyts E , Frydelund‐Larsen L , Quintana‐Murci L , McElreavey K , Skakkebaek NE . Double‐blind Y chromosome microdeletion analysis in men with known sperm parameters and reproductive hormone profiles: microdeletions are specific for spermatogenic failure. J Clin Endocrinol Metab. 2001;86:2638‐2642. 11397865 10.1210/jcem.86.6.7527

[2] Simoni M , Tüttelmann F , Gromoll J , Nieschlag E . Clinical consequences of microdeletions of the Y chromosome: the extended Münster experience. Reprod Biomed Online. 2008;16:289‐303. 18284889 10.1016/s1472-6483(10)60588-3

[3] Lo Giacco D , Chianese C , Sanchez‐Curbelo J , et al. Clinical relevance of Y‐linked CNV screening in male infertility: new insights based on the 8‐year experience of a diagnostic genetic laboratory. Eur J Hum Genet. 2014;22:754‐761. 24193344 10.1038/ejhg.2013.253 PMC4023226

[4] Vogt PH , Edelmann A , Kirsch S , et al. Human Y chromosome azoospermia factors (AZF) mapped to different subregions in Yq11. Hum Mol Genet. 1996;5:933‐943. 8817327 10.1093/hmg/5.7.933

[5] Foresta C , Ferlin A , Moro E . Deletion and expression analysis of AZFa genes on the human Y chromosome revealed a major role for DBY in male infertility. Hum Mol Genet. 2000;9:1161‐1169. 10767340 10.1093/hmg/9.8.1161

[6] Sun C , Skaletsky H , Rozen S , et al. Deletion of azoospermia factor a (AZFa) region of Y chromosome caused by recombination between HERV15 proviruses. Hum Mol Genet. 2000;9:2291‐2296. 11001932 10.1093/oxfordjournals.hmg.a018920

[7] Kamp C , Huellen K , Fernandes S , et al. High deletion frequency of the complete AZFa sequence in men with Sertoli‐cell‐only syndrome. Mol Hum Reprod. 2001;7:987‐994. 11574668 10.1093/molehr/7.10.987

[8] Fernandes S , Huellen K , Goncalves J , et al. High frequency of DAZ1/DAZ2 gene deletions in patients with severe oligozoospermia. Mol Hum Reprod. 2002;8:286‐298. 11870237 10.1093/molehr/8.3.286

[9] Costa P , Gonçalves R , Ferrás C , et al. Identification of new breakpoints in AZFb and AZFc. Mol Hum Reprod. 2008;14:251‐258. 18326547 10.1093/molehr/gan014

[10] Kleiman SE , Yogev L , Lehavi O , et al. The likelihood of finding mature sperm cells in men with AZFb or AZFb‐c deletions: six new cases and a review of the literature (1994–2010). Fertil Steril. 2011;95:2005‐2012. 21367410 10.1016/j.fertnstert.2011.01.162

[11] Soares AR , Costa P , Silva J , Sousa M , Barros A , Fernandes S . AZFb microdeletions and oligozoospermia‐which mechanisms? Fertil Steril. 2012;97:858‐863. 22317821 10.1016/j.fertnstert.2012.01.099

[12] Kent‐Firs MG , Kol S , Muallem A , et al. The incidence and possible relevance of Y‐linked microdeletions in babies born after intracytoplasmic sperm injection and their infertile fathers. Mol Hum Reprod. 1996;2:943‐950. 9237238 10.1093/molehr/2.12.943

[13] Mulhall JP , Reijo R , Alagappan R , et al. Azoospermic men with deletion of the DAZ gene cluster are capable of completing spermatogenesis: fertilization, normal embryonic development and pregnancy occur when retrieved testicular spermatozoa are used for intracytoplasmic sperm injection. Hum Reprod. 1997;12:503‐508. 9130751 10.1093/humrep/12.3.503

[14] Oliva R , Margarit E , Ballescá JL , et al. Prevalence of Y chromosome microdeletions in oligospermic and azoospermic candidates for intracytoplasmic sperm injection. Fertil Steril. 1998;70:506‐510. 9757880 10.1016/s0015-0282(98)00195-2

[15] Hopps CV , Mielnik A , Goldstein M , Palermo GD , Rosenwaks Z , Schlegel PN . Detection of sperm in men with Y chromosome microdeletions of the AZFa, AZFb and AZFc regions. Hum Reprod. 2003;18:1660‐1665. 12871878 10.1093/humrep/deg348

[16] Stahl PJ , Masson P , Mielnik A , Marean MB , Schlegel PN , Paduch DA . A decade of experience emphasizes that testing for Y microdeletions is essential in American men with azoospermia and severe oligozoospermia. Fertil Steril. 2010;94:1753‐1756. 19896650 10.1016/j.fertnstert.2009.09.006

[17] Mateu E , Rodrigo L , Martínez MC , et al. Aneuploidies in embryos and spermatozoa from patients with Y chromosome microdeletions. Fertil Steril. 2010;94:2874‐2877. 20655521 10.1016/j.fertnstert.2010.06.046

[18] Van Golde RJ , Wetzels AM , de Graaf R , Tuerlings JH , Braat DD , Kremer JA . Decreased fertilization rate and embryo quality after ICSI in oligozoospermic men with microdeletions in the azoospermia factor c region of the Y chromosome. Hum Reprod. 2001;16:289‐292. 11157822 10.1093/humrep/16.2.289

[19] Krausz C , Hoefsloot L , Simoni M , Tüttelmann F . EAA/EMQN bestpractice guidelines for molecular diagnosis of Y‐chromosomal microdeletions: state‐of the art 2013. Andrology. 2014;2:5‐19. 24357628 10.1111/j.2047-2927.2013.00173.x PMC4065365

[20] Schlegel PN . Testicular sperm extraction: microdissection improves sperm yield with minimal tissue excision. Hum Reprod. 1999;14:131‐135. 10374109 10.1093/humrep/14.1.131

[21] Huirne JA , Homburg R , Lambalk CB . Are GnRH antagonists comparable to agonists for use in IVF? Hum Reprod. 2007;22:2805‐2813. 17872909 10.1093/humrep/dem270

[22] Pinto F , Oliveira C , Cardoso MF , et al. Impact of GnRH ovarian stimulation protocols on intracytoplasmic sperm injection outcomes. Reprod Biol Endocrinol. 2009;7:5. 19146685 10.1186/1477-7827-7-5 PMC2633006

[23] Kostiner DR , Turek PJ , Reijo RA . Male infertility: analysis of the markers and genes on the human Y chromosome. Hum Reprod. 1998;13:3032‐3038. 9853850 10.1093/humrep/13.11.3032

[24] Krausz C , Degl'Inocenti S . Y chromosome and male infertility: update. Front Biosci. 2006;11:3049‐3061. 16720375 10.2741/2032

[25] Ferlin A , Arredi B , Speltra E , et al. Molecular and clinical characterization of Y chromosome microdeletions in infertile men: a 10‐year experience in Italy. J Clin Endocrinol Metab. 2007;92:762‐770. 17213277 10.1210/jc.2006-1981

[26] Longepied G , Saut N , Aknin‐Seifer I , et al. Complete deletion of the AZFb interval from the Y chromosome in an oligozoospermic man. Hum Reprod. 2010;25:2655‐2663. 20716563 10.1093/humrep/deq209

[27] Brandell RA , Mielnik A , Liotta D , et al. AZFb deletions predict the absence of spermatozoa with testicular sperm extraction: preliminary report of a prognostic genetic test. Hum Reprod. 1998;13:2812‐2815. 9804236 10.1093/humrep/13.10.2812

